# The Corn Doctor’s Progress

**Published:** 1879-07

**Authors:** Edmund Andrews

**Affiliations:** Chicago, No. 6 Sixteenth St.


### ARTICLE III.

### THE CORN DOCTOR'S PROGRESS. By EDMUND ANDREWS, A.M., M.D..

Those who remember the humble but rascally corn-doctor of yore, who aspired to nothing higher than a simultaneous shaving of the corns and the purses of his customers, may learn that this despised business is undergoing a change, and that it bids fair to become as honest and legitimate a specialty as dentistry.

Some readers will remember one of the old stock who appeared years ago in Chicago, with a sign 3 feet wide and 25 feet long, lettered something as follows:

JOHN DOE, CHIROPODIST,
ANATOMICAL PROFESSOR OF THE PATHOLOGICAL DISEASES OF THE HUMAN FOOT.

He published pretended letters from Queen Victoria and Napoleon III., thanking him for the amazing talent and "grande habileté" with which he had pared their imperial corns, and charged ten dollars a corn for extraction, with a guaranty of permanent cure. He had a trick of shaving off the callosity in successive layers, and charging each layer as a separate ten dollar corn. In this way he found on one astonished Chicago gentleman nine corns on one spot, and in a few minutes delighted him by presenting a bill of ninety dollars. He took care to leave town before the corns had time to grow again.

This is a prosaic age, and such magnificent specimens of impudence appear no more among us. The enterprising corn-doctor has departed, and in his stead appears the modern chiropodist, who goes quietly and honestlv about his work, like other common mortals.

We have in Chicago five of them. They make no secret of their methods, and I had no difficulty in collecting the following information. They say with one voice that there is no literature of any consequence on the subject, and that they are obliged to learn the business from each other without the aid of books. In this, however, they are partly mistaken, for a good deal has been printed on the subject, but it must be confessed that most of it is of a wretchedly poor quality, and of such a contradictory character, that the less it is read, the less it will confuse the student. All text books on skin diseases and general surgery touch upon the subject, and there are numerous treatises and articles about it in French and German, but the absurd and contradictory remarks let slip by some of the most eminent authors are discreditable to our profession.

The Chicago chiropodists seem for the most part to be pursuing their business in an honest and reputable manner, and some of them have the very best families of the city and of the country among their patrons. They are rising by degrees towards the rank of a legitimate and honorable specialty, though at present they are decidedly lacking in the amount of education which they ought to possess.

Their work is upon corns, bunyons and ingrowing nails. For extracting corns the price is from half a dollar to a dollar each, and they take care of patients by the year for $10 or $20.

They have the same obscure and contradictory ideas about the structure of corns, which exist among authors, especially on the question whether there are originally any living papillæ running up through the central spike or "*racine*" of the corn. I have not learned of any investigations among them to settle the point, other than to note the little blood clots occasionally enclosed in the "racine."

Their instruments are simple. One family of chiropodists, which has branches in New York, Boston, St. Louis and Chicago, use mostly small, thin chisels, with the edges running obliquely across the blade. This is handled with a lateral movement, while a much narrower kind is used to work around and lift out the central root. Some instruments have round ends for working between the toes. After extraction the operator cuts a hole in a small piece of thick buckskin, and applies it over the spot by the help of adhesive plaster, and they thus relieve the patient for two or three months.

They all agree, of course, that faulty shoes are the cause of the corns, and their uniform direction for correct ones is to make the heels low, so that the foot shall not slide down upon the toes; to have it snug on the instep to hold the foot back, and to make the toes wide and long. One of them has a shop attached to his office, where he superintends the construction of proper shoes for his patrons.

A chiropodist named Willard has an operation for ingrowing nail, which may be original; at any rate, I have not yet found it in any book. He asserts, what is pretty nearly true, that the term ingrowing nail is a misnomer, the nail itself being unchanged in form. It is simply an overgrowing of the flesh, with inflammation. He neither extracts the nail nor slices off the overlapping flesh, but cuts out a narrow ellipse of tissue near the nail and parallel to its border, claiming that the border itself, where it rests against the edge of the nail, has its special structure adapted to its location, and ought not to be sacrificed. The removal of the strip of flesh being accomplished, he brings the edges of the wound together with fine sutures, thus drawing the border away from the nail, and effecting a cure. I have not yet tried the plan, but it seems worthy of being tested.

When the chiropodists have made a further advance in education, they will probably add the treatment of talipes to their work, but at present they are neither competent for it nor attempt it, except in a few instances.

They are now in a transition state, but we may hope in time to see them become a well-educated class of men.

### BIBLIOGRAPHY.

As before stated, the literature of this subject is in a scattered form, so that the chiropodists generally are unaware of its existence. The following references cover most of it:

Anandale, on Malformations of Fingers and Toes; Edinburg.

Anandale, on Corns and Bunyons; London.

Chapters and paragraphs in text-books on skin diseases and general surgery, viz. : Tilbury Fox, Neumann, Duhring and Wilson on Skin Diseases ; Cooper's Surg. Dictionary, Brodie's Clinical Lectures, Cruveilhier's Path. Anat., Syme's Surgical Works by McLean, and the surgical text-books of Holmes, Gant, Bryant, Gross, Ashurst, Erichsen, Druitt, Stromeyer, Pithlea and Billroth, Velpeau and Malgaigne.

The Transactions of the Am. Med. Assn. contain brief articles on the subject in various places.

Follin's chapter in his Pathologie Externe : tome 1, 1866, is specially valuable.

There are articles in the Dictionaire de Thérapeut. Méd. et Chirurg., Dictionaire des Sciences-Méd., and Nouveau Dict. de Méd. et Chir.

The following writers touch the subject in monographs or in more formal works :

Roche et Sanson, Pathologie Medico-Chir.

Wattelbled, Bulletin Acad. Méd., Paris, 1836–7, tome I.

Ruchert, De Clavo Nonnulla, Berlin, 1846.

Boyer, Des Cors aux Pieds., 1847.

Jolieu, Des Tum. Chir. de la Peau, Thèse Inaug., Paris, 1855.

Gorju, Observations, etc., Thèse Inaug., Paris, 1857.

Rindfleisch, Traité d' Histol. Path., 1875.

Bouchardat, Nouvelles Formules Magistrales, 1876.

The following are a few of the more important articles in the older journals :

*Journal de Vandermonde, juillet*, p. 88, 1762.

" " " p. 449, 1775.

*Berlin Hosp. Gazette*, p. 516, 1844.

*Med. Times*, Sept. 4, 1852.

*Monit. des Hôpitaux*, 1856.

CHICAGO, NO. 6 SIXTEENTH ST., May 1, 1879.

---

A YOUNG, active and well educated physician, regular practice, can learn of an opportunity of securing a professional income amounting to between two and three thousand per annum, with a prospect of increasing this amount, by applying to Dr. J. H. Etheridge, 603 Michigan avenue, Chicago.

